# Correction: Ligand Binding at the α4-α4 Agonist-Binding Site of the α4β2 nAChR Triggers Receptor Activation through a Pre-Activated Conformational State

**DOI:** 10.1371/journal.pone.0165184

**Published:** 2016-10-19

**Authors:** The PLOS ONE Staff

## Notice of Republication

This article was republished on September 8, 2016, to correct errors in the article title that were introduced during the typesetting process. The publisher apologizes for the errors. Please download this article again to view the correct version. The originally published, uncorrected article and the republished, corrected article are provided here for reference.

## Supporting Information

S1 File Originally published, uncorrected article. (PDF)

S2 File Republished, corrected article. (PDF)
