# Supplementary material for: Ligand Binding at the α4-α4 Agonist-Binding Site of the α4β2 nAChR Triggers Receptor Activation through a Pre-Activated Conformational State
Source: PLoS One. 2016 Aug 23;11(8):e0161154. doi: 10.1371/journal.pone.0161154 (PMC4995024; doi:10.1371/journal.pone.0161154)
Supplement: S1 Fig — Effect of Changes in Est Pomax on concentration-response relationships derived from both A the desensitizing (Model 1) and B the pre-activated model (Model 2). Peak currents to Saz-A (●) and without (○) 10 μM NS-9283, and to TC-2559 with (■) and without (□) 10 μM NS9283 were normalized to an Est Pomax for 1 mM ACh of 0.4 (left) and 0.45 (right). Dashed lines represent the simulated curves from A Model 1 where NS9283 alters the gating constant E, and B Model 2 where either the pre-activated constant F is altered (CF.F) or the gating constant (CE.E) is altered by NS9283. (PDF) [file pone.0161154.s001.pdf]

Supporting Figure S1.

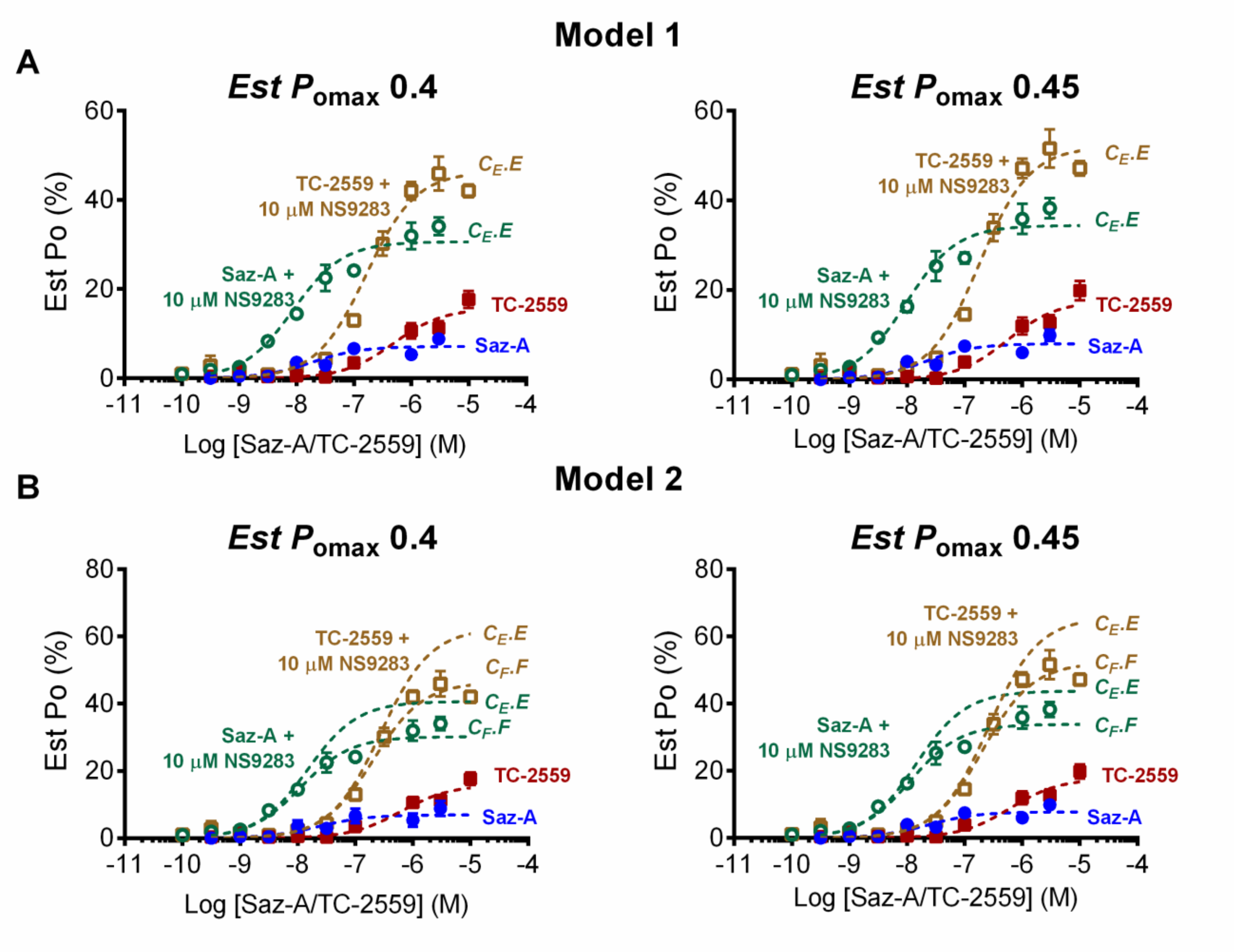

Supporting Figure S1. Effect of Changes in *Est* P_{omax} on concentration-response relationships derived from both **A** the desensitizing (Model 1) and **B** the pre-activated model (Model 2). Peak currents to Saz-A (●) and without (○) 10 μM NS-9283, and to TC-2559 with (■) and without (□) 10 μM NS9283 were normalized to an *Est* P_{omax} for 1 mM ACh of 0.4 (left) and 0.45 (right). Dashed lines represent the simulated curves from A Model 1 where NS9283 alters the gating constant E, and **B** Model 2 where either the pre-activated constant F is altered ($C_F.F$) or the gating constant ($C_{E}.E$) is altered by NS9283.
