# Supplementary material for: Correction: Ligand Binding at the α4-α4 Agonist-Binding Site of the α4β2 nAChR Triggers Receptor Activation through a Pre-Activated Conformational State
Source: PLoS One. 2016 Oct 19;11(10):e0165184. doi: 10.1371/journal.pone.0165184 (PMC5070942; doi:10.1371/journal.pone.0165184)
Supplement: S1 File — (PDF) [file pone.0165184.s001.PDF]

PLOS ONE

RESEARCH ARTICLE

Ligand Binding at the 4-4 Agonist-Binding Site of the 42 nAChR Triggers Receptor Activation through a Pre-Activated Conformational State

Dinesh C. Indurthi[1], Trevor M. Lewis[2], Philip K. Ahring[1], Thomas Balle[1], Mary Chebib[1]*, Nathan L. Absalom[1]*

1 Faculty of Pharmacy, University of Sydney, NSW, 2006, Australia, **2** School of Medical Sciences, University of NSW, Kensington, NSW, 2052, Australia

* nathan.absalom@sydney.edu.au (NLA); mary.collins@sydney.edu.au (MC)

Abstract

The α4β2 nicotinic acetylcholine receptor (nAChR) is the most abundant subtype in the brain and exists in two functional stoichiometries: $(\alpha4)_3(\beta2)_2$ and $(\alpha4)_2(\beta2)_3$. A distinct feature of the $(\alpha4)_3(\beta2)_2$ receptor is the biphasic activation response to the endogenous agonist acetylcholine, where it is activated with high potency and low efficacy when two α4-β2 binding sites are occupied and with low potency/high efficacy when a third α4-α4 binding site is occupied. Further, exogenous ligands can bind to the third α4-α4 binding site and potentiate the activation of the receptor by ACh that is bound at the two α4-β2 sites. We propose that perturbations of the recently described pre-activation step when a third binding site is occupied are a key driver of these distinct activation properties. To investigate this, we used a combination of simple linear kinetic models and voltage clamp electrophysiology to determine whether transitions into the pre-activated state were increased when three binding sites were occupied. We separated the binding at the two different sites with ligands selective for the α4-β2 site (Sazetidine-A and TC-2559) and the α4-α4 site (NS9283) and identified that when a third binding site was occupied, changes in the concentration-response curves were best explained by an increase in transitions into a pre-activated state. We propose that perturbations of transitions into a pre-activated state are essential to explain the activation properties of the $(\alpha4)_3(\beta2)_2$ receptor by acetylcholine and other ligands. Considering the widespread clinical use of benzodiazepines, this discovery of a conserved mechanism that benzodiazepines and ACh potentiate receptor activation *via* a third binding site can be exploited to develop therapeutics with similar properties at other cys-loop receptors.

OPEN ACCESS

Citation: Indurthi DC, Lewis TM, Ahring PK, Balle T, Chebib M, Absalom NL (2016) Ligand Binding at the 4-4 Agonist-Binding Site of the 42 nAChR Triggers Receptor Activation through a Pre-Activated Conformational State. PLoS ONE 11(8): e0161154. doi:10.1371/journal.pone.0161154

Editor: Thomas Arendt, Universitatsklinikum Leipzig, GERMANY

Received: April 20, 2016

Accepted: August 1, 2016

Published: August 23, 2016

Copyright: © 2016 Indurthi et al. This is an open access article distributed under the terms of the Creative Commons Attribution License, which permits unrestricted use, distribution, and reproduction in any medium, provided the original author and source are credited.

Data Availability Statement: All relevant data are within the paper and its Supporting Information Files.

Funding: This work was funded by the National Health and Medical Research Council of Australia (NHMRC) Project Grant 1069417 (https://www.nhmrc.gov.au/grants-funding/apply-funding/project-grants). DCI is funded by an International Postgraduate Research Scholarship by the University of Sydney (http://sydney.edu.au/scholarships/prospective/international_postgraduate_scholarships.shtml#iprs) and received support of a John Lamberton Scholarship

Introduction

Nicotinic acetylcholine receptors (nAChRs) are members of the cys-loop receptor superfamily of ligand-gated ion channels (LGICs) and form a pentameric subunit arrangement around a

(http://agile2.ucc.usyd.edu.au/ro/opportunities/scholarships/1260). This work was co-funded by Imk Almene Fond, Denmark and Saniona A/S (http://saniona.com/EN/).

Competing Interests: The authors have declared that no competing interests exist.

cation-conducting central ion pore. Once activated by acetylcholine (ACh) in the CNS, nAChRs are involved in initiation of action potentials to modulate vesicular neurotransmitter release that activates post-synaptic receptors [1,2]. The heteromeric α4β2 is the most abundant nAChR subtype in central nervous system along with the homomeric α7 receptor and is considered to be an important target for treating numerous neurological impairments including schizophrenia, attention deficit hyperactivity disorder (ADHD) and pain [3–6].

The α4β2 nAChR forms two distinct receptors that differ in subunit stoichiometry, $(\alpha4)_3(\beta2)_2$ and $(\alpha4)_2(\beta2)_3$ (or 3α4:2β2 and 2α4:3β2, receptors, respectively) [7]. The two stoichiometries have distinct agonist sensitivities [8,9], receptor desensitization[10], agonist affinity [11], Ca^{2+} permeability [12] and have different pharmacology for several ligands [13–15]. This has been shown to be due to the presence of an additional agonist binding site (α4-α4) in the 3α4:2β2 stoichiometry that is ~100 fold less potent (for ACh) than the conventionally known agonist binding site (α4-β2) that is present in both stoichiometries [16,17]. As a result the 3α4:2β2 receptor gives a biphasic activation curve to ACh, whereby the receptor is activated with high levels of efficacy when three molecules of ACh are bound (at α4-α4 and α4-β2 interfaces), and with low levels of efficacy when two molecules of ACh are bound (at α4-β2 interfaces) [11,17,18]. Further, ligands that bind only at the α4-β2 interfaces, including Sazetidine-A and TC-2559 activate the receptor while ligands that bind only at the α4-β2 interfaces, such as NS9283, enhance the activation of the receptor by ACh without activating the native receptor [14,19].

We have shown that three residues, H142, Q150, and T152 on the complimentary side of the α4 subunit and V136, F144, and L146 on the corresponding side of the β2 subunit, constitute the core difference between the two interfaces [17]. We used this information to show the modulator NS9283 that selectively binds at the α4-α4 interface had an agonist-like mechanism by activating *via* this interface [20]. We further determined the differences in binding affinities between the two sites [11] and studied structure activity relation (SAR) for ligands that have different pharmacological profiles on the two stoichiometries [21]. However, the transitions of the receptor during activation that are perturbed when two, or three, agonist-binding sites are occupied are not understood, along with the transitions that are perturbed by modulators that potentiate receptor activation.

Agonist binds at the extracellular domain to initiate a series of conformational changes leading to opening of the channel pore some 50 Å away at the transmembrane domain (TM2). This allosteric receptor activation can be described by kinetic models that define discrete, multiple transition states that are too short to be captured by spectroscopic or crystallographic techniques [22,23]. The classic three-state kinetic model of *del Castillo-Katz* that first described agonist activation included a closed, agonist bound and open state [24]. A further closed state subsequent to channel opening, the desensitized state, was introduced to explain receptor closure as a result of prolonged agonist exposure [25,26]. Subsequently, a considerable body of work has been directed towards understanding the receptor transitions from the agonist-bound closed to open states. Φ-value analysis of the rate-equilibrium linear free-energy relationships of a series of mutations gave the relative timing of the movement for particular residues in the 'conformational wave' of receptor activation, with different regions of the protein changing conformation in a step-wise process from ligand binding to channel opening [23]. A recent significant advance was the detection of intermediate closed states between ligand binding and channel opening called 'prime' and 'flip' states [27,28]. The 'flip' mechanisms describes a concerted conformational change among all subunits within the receptor complex, whereas a 'priming' mechanism describes localised conformational changes at the binding site that initiates channel gating. Transitions into these pre-activated states were found to be crucial in

defining the efficacy of an agonist, giving a framework to explain how partial agonists activate receptors with low efficacy.

Modulators of cys-loop receptors include many classes of compounds that are used either clinically or in research, including barbiturates and benzodiazepines that modulate $GABA_A$Rs, and compounds such as estradiol and PNU-120596 that modulate nAChRs. These modulators bind to the receptor complex and, in combination with an agonist, alter the conformational transitions between states to ultimately favour an open state, potentiating the response to the agonist. To achieve this, different steps in the activation pathway can be altered by the modulator depending on the location of their binding site and the conformational change induced to the receptor. For instance, PNU-120596 is a positive modulator of α7 nAChRs that primarily decreases transitions into the desensitized state [29], while diazepam modulates $GABA_A$Rs by increasing transitions into a pre-activated state [30]. These different mechanisms of modulation lead to differences in the pharmacological properties that underlie the physiological response to a modulator.

Typically, activation of Cys-loop receptors has been studied by developing kinetic models of activation and fitting data from high-resolution single-channel recordings to these models. Acquiring single-channel recordings from α4β2 nAChRs has been difficult, but an alternative method using simpler linear models and whole-cell electrophysiological recordings has recently been described to explain how benzodiazepines potentiate GABA activation of $GABA_A$Rs through binding at a third site homologous to the GABA-binding site [30]. Similar to benzodiazepines, both ACh and NS9283, a ligand that augments cognitive function in rodents, bind at the third α4-α4 binding site of 3α4:2β2 nAChRs to potentiate receptor response, and may increase receptor activation via a similar mechanism.

Here we delineate currents elicited from α4-β2 (Sazetidine-A or TC-2559) and α4-α4 (NS9283) activation. By developing two linear kinetic models that can differentiate the transitions between receptor states, we show that the increased efficacy of activation when a third agonist (NS9283) is bound is due to a shift in the equilibrium from the ligand-bound closed state to a pre-activated or "flip" state. We propose that ligands that bind at a third homologous binding site will potentiate receptor activation by the same mechanism.

Materials and Methods

Molecular Biology

cDNA encoding human nAChR α4 and β2 subunits were cloned, and cRNA was prepared as previously described [31]. A triple-point-mutated $β2^m$ subunit with the mutations V136H, F144Q, and L146T was constructed as previously described [17].

Oocyte Preparation and Electrophysiology

This study was carried out in strict accordance with the Australian Code of Practice for the Care and Use of Animals for Scientific Purposes. The protocol for this specific study was approved by the Animal Ethics Committee of the University of Sydney (Protocol number: 2013/5915). All surgical procedures were carried out in under anesthesia induced by immersion in a tricaine solution to ameliorate suffering. All *Xenopus* were monitored during surgery and for the following 5–7 days to ensure both anesthesia and the surgery did not cause adverse effects. Mature female *Xenopus laevis* frogs were anaesthetised with 0.17% tricaine (buffered with 0.06% sodium bicarbonate) for 15 minutes, after which the loss of righting reflex was confirmed before transferring on to ice where surgeries were performed. A small (1–2 cm) abdominal incision was made through both the skin and muscle layer with surgical knives. Ovary lobes were removed with a pair of forceps, and kept in oocyte releasing 2 (OR2) buffer (82.5

mM NaCl, 2 mM KCl, 1 mM $MgCl_2$, 5 mM HEPES hemisodium; pH 7.4). The skin and muscle layer were sutured separately, and frogs were allowed to recover for six months before they were reselected for surgeries. A total of five recoverable surgeries with 6–12 months recovery periods between surgeries were performed on each frog to reduce the adverse effects of *Xenopus* transportation into Australia, as approved by the Animal Ethics Committee of the University of Sydney (Protocol number: 2013/5915) before a terminal surgery was performed, in which a lethal dose of tricaine (0.5%) was used. Briefly, lobes from ovaries of female adult *X. laevis* were removed and defolliculated to obtain isolated oocytes. Oocytes were injected with a total of ∼25 ng of cRNA encoding human WT receptor as shown earlier to express 3α4:2β2 and 2α4:3β2 [17], and incubated for 2–5 days at 15–18°C.

Oocytes were subjected to two-electrode voltage-clamp electrophysiological testing using a custom-built system where solutions were applied directly to the oocytes via a glass capillary tube placed in the vicinity of the cell. This ensured a solution exchange rate faster than two seconds. Ligand was applied for 30 sec and peak current amplitudes were measured. To minimise the effects of long desensitized states, the number of applications of ligand at each oocyte was minimized rather than performing full concentration-response curves on individual oocytes.

Data Analysis

Concentration–response curves for Sazetidine-A and TC-2559 were fitted using GraphPad Prism 6 to a monophasic Hill equation with Hill Slope of 1:

$$I = I_{\max}\left(\frac{1}{1+\left(\frac{\mathrm{EC50}}{[\mathrm{A}]}\right)}\right)$$

Where I_{max} is the maximum current, EC_{50} is the concentration that produces the half-maximal response and [A] is the concentration of ligand.

Concentration–response curves for ACh were fitted using GraphPad Prism 6 to a biphasic Hill equation with Hill slopes of 1:

$$I = I_{\max} * \left(\left(\frac{\mathrm{Frac1}}{1+\left(\frac{\mathrm{EC50(1)}}{[\mathrm{A}]}\right)}\right)+\left(\frac{\mathrm{Frac2}}{1+\left(\frac{\mathrm{EC50(2)}}{[\mathrm{A}]}\right)}\right)\right)$$

Where Frac1 and Frac2 are the fractions and $EC_{50(1)}$ and $EC_{50(2)}$ are the half-maximal concentrations of high and low-sensitivity phases of the concentration-response curves, respectively.

Results

To understand how additional ligand binding at the α4-α4 site enhances receptor activation compared to when only two α4-β2 sites are occupied, we used simple linear kinetic models to interpret the data. This requires ligands that selectively bind at the α4-β2 and α4-α4 sites to activate the receptor.

Saz-A and TC-2559 Selectively Activate via the α4-β2 Interface

To determine if Saz-A and TC-2559 were indeed activating the receptor exclusively through binding at the α4-β2 sites, concentration-response relationships (CRR) of Saz-A and TC-2559 were constructed for wild-type (WT) 2α4:3β2 and 3α4:2β2 receptors. Saz-A was a full agonist and TC-2559 acted as a super agonist at 2α4:3β2 receptors with maximum responses of 110% and 360%, respectively, when normalised to the response of a saturating concentration of ACh

(1 mM), although the maximum response to TC-2559 was difficult to estimate as the top of the concentration-response curve may not have been reached (Table 1, Fig 1). However, Saz-A and TC-2559 were both partial agonists at 3α4:2β2, eliciting a maximum response of 19% and 49% respectively, (Table 1, Fig 1D), consistent with these molecules binding at only two α4-β2 sites to activate the receptor, in comparison to ACh that binds at three (two α4-β2 and an α4-α4). Furthermore, both ligands exhibited similar potencies at 3α4:2β2 and 2α4:3β2 receptors with EC_{50} values of 23 and 10 nM for Saz-A and a 5-fold difference with EC_{50} values of 3300 and 642 nM for TC-2559, respectively, (Table 1) suggesting that both receptors were activated via identical sites. Both Saz-A and TC-2559 do not seem to affect the recovery of the current after application (Fig 1B and 1C).

Nevertheless, a contribution to Saz-A and TC-2559 activation from binding at the α4-α4 interface cannot be excluded from these experiments. Therefore, Saz-A and TC-2559 were applied to mutated 3α4:2β2^{HQT} receptors where the α4-β2 binding site is abolished. The β2^{HQT} construct was designed such that the complementary (-) side of the agonist binding pocket resembled the α4(-) face so that the receptors contain only α4-α4-like binding sites [11,17,32]. Neither Saz-A or TC-2559 activated 3α4:2β2^{HQT} mutant receptors (Fig 1D and 1E; Table 1), demonstrating that Saz-A and TC-2559, at the concentrations tested, bind only at α4-β2 interfaces to activate 3α4:2β2 nAChRs and can therefore be used to mimic ACh activation when two α4-β2 binding sites are occupied.

Delineating 3α4:2β2 Receptor Activation from α4-β2 and α4-α4 Binding Interfaces

NS9283 selectively binds to the α4-α4 binding site to increase receptor activation [6]. 3α4:2β2 receptor activation from α4-β2 and α4-α4 binding interfaces can then be obtained by co-

Table 1. Parameters derived from fitting of data to the Hill Equation with a Hill co-efficient of one.

Receptor	Ligand(s)	EC_{50}[1] (nM) (95% CI)	$I/I_{1mM\ ACh}$[2] (%)	Est P_{omax}[3] (%)	N[4]
3α4:2β2	ACh			51 ± 5	7
	Saz-A	23	18 ± 1.4	9 ± 0.7	6
		(11–46)			
	TC-2559	640	41 ±4	20 ± 2	6
		(270–1500)			
	Saz-A +	11	80 ± 5	39 ± 1.5	6
	NS9283 (10μM)	(8–16)			
	TC-2559+	200	120 ± 3	59 ± 1.5	6
	NS9283 (10μM)	(154–255)			
2α4:3β2	Saz-A	10	108 ± 6	-	4
		(5.47–19.7)			
	TC-2559	3300	330 ± 10	-	4
		(2600–4200)			
3α4:2β2^{HQT}	Saz-A	-	0*	-	2
	TC-2559	-	0*	-	3

Experimentally derived values are obtained from concentration-response relationship curve fitted to a hill co-efficient of one using prism 6.

[1]Mean EC_{50s} values and 95% confidence interval derived from the curve-fitting are shown in the bracket.

[2]Mean I_{max} normalized 1 mM ACh with ± SEM derived from the curve-fitting is shown.

[3]Estimated $P_{o,max}$ ± SEM is obtained by normalizing with 1 mM ACh + NS206 (10 μM).

[4]As desensitization rates of Saz-A and TC-2559 were high, the N refers to the number of replicates at each data point, rather than the number of individual concentration-response curves.

doi:10.1371/journal.pone.0161154.t001

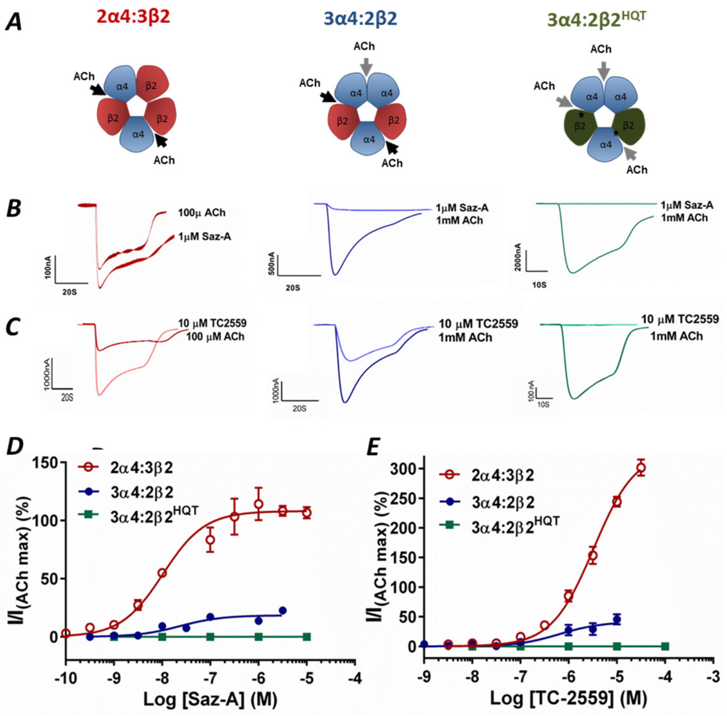

Fig 1. Saz-A and TC-2559 act selectively at the α4-β2 interface. A Schematic showing α4-β2 (black arrow) and α-α (grey arrow) binding sites for ACh at the 3α4:2β2, 2α4:3β2 and 3α4:2β2^{HQT} receptors. **B.** Current traces of Saz-A (1μM) and **C.** TC-2559 (10μM) for 3α4:2β2, 2α4:3β2 and 3α4:2β2^{HQT} receptor compared to maximum ACh currents on the same oocyte. Peak currents elicited by **D** Saz-A and **E** TC-2559 were normalized to the saturating concentration of ACh and fitted to non-linear curve fits. The injection ratios of α4:β2 that correspond to the 3α4:2β2 (blue) and the 2α4:3β2 (red) receptors are 4:1 and 1:4 respectively. Saz-A and TC-2559 do not activate the 3α4:2β2^{HQT} (green) receptor that has three α4-α4-like interfaces. Dots represent the mean ± s.e.m.

doi:10.1371/journal.pone.0161154.g001

applying NS9283 with Saz-A or TC-2559 (Fig 2). Co-application of Saz-A (1 μM) and TC-2559 (10 μM) with NS9283 (10 μM) had peak currents comparable to max ACh (1 mM) application, with no significant effect on current recovery after drug application that suggests no effect on desensitization (Fig 2B and 2C). NS9283 potentiation curves were generated by co-applying 10 μM NS9283 with varying concentrations of Saz-A and TC-2559 to WT 3α4:2β2 nAChRs (Fig 2D and 2E). For Saz-A, co-application of NS9283 (10 μM) increased the maximum response by 4.4-fold and decreased the EC_{50} value by 2-fold (Table 1), while for TC-2559 the maximum response was increased by 2.9-fold and the EC_{50} value was decreased 3.1-fold (Table 1). Thus, NS9283 potentiation of Saz-A or TC-2559 resulted primarily in an increased efficacy of receptor activation, that is comparable to the increase observed when a third molecule of ACh binds to the α4-α4 interface. Therefore, we used Saz-A or TC-2559 in combination with NS9283 to determine how binding at the third α4-α4 site increases activation levels of the receptor.

Estimating Maximum Open Probability

Ideally, single-channel recordings would be used to estimate the $P_{o,max}$ values; however this is technically highly challenging for α4β2 nAChRs due to channel rundown and desensitization [33]. To overcome this, an alternate method utilizing co-application of ACh and NS206, a positive allosteric modulator (PAM), was used to estimate the maximum open probability (*Est.* $P_{o,max}$)

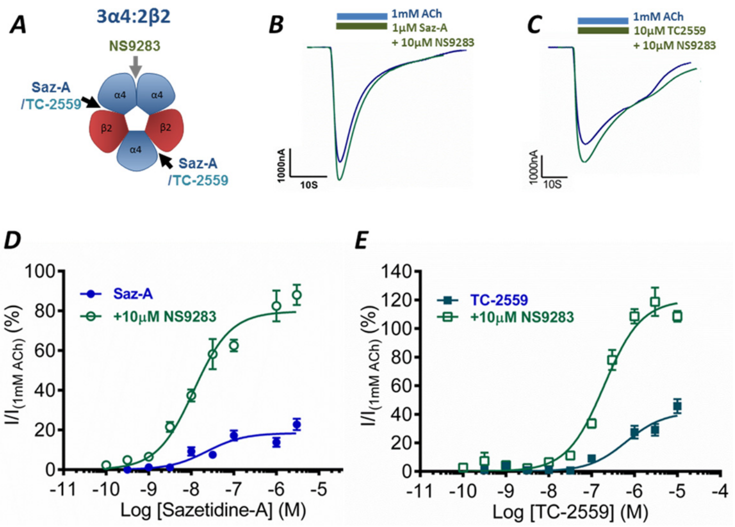

Fig 2. Saz-A and TC-2559 co-application with NS9283: **A** Schematic of the 3α4:2β2 receptor with the binding sites of Saz-A and TC-2559 at the α4-β2 interface, and NS9283 at the α4-α4 interface. Representative current trace of ***B.*** Saz-A and ***C.*** TC-2559, co-applied with NS9283 (10μM) (Green) compared to max ACh (1mM) current on 3α4:2β2 receptors ***D*** Saz-A and ***E*** TC-2559 concentration-response curves in the absence (blue) and presence (green) of 10 μM NS9283 normalized to 1 mM ACh at 3α4:2β2 receptors. Dots represent the mean ± s.e.m.

doi:10.1371/journal.pone.0161154.g002

at 3α4:2β2 receptors. NS206 binds at an alternative site to NS9283, increasing the maximum response of the receptor above the maximum response to ACh [19,34]. We have assumed that when both the positive allosteric modulator and native ligand ACh are bound, the probability of the receptor being opened is approaching one, and was previously successfully used for other members of LGICs [35]. All other responses are then expressed relative to this maximum response (Fig 3).

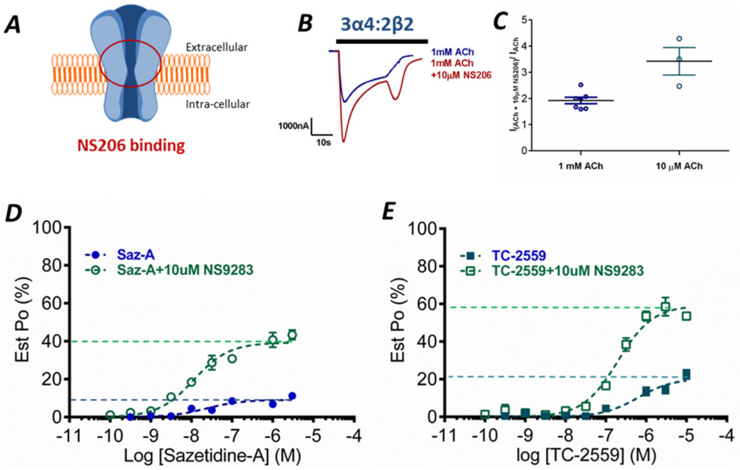

Fig 3. Estimation of open probability. ***A*** Schematic depicting the hypothesized areas for NS206 binding [34]. ***B*** Representative current trace of the response of the 3α4:2β2 receptor to 1 mM ACh (blue) and 1 mM ACh co-applied with 10 μM NS206 to estimate open channel probability. ***C*** The level of NS206 (10μM) positive modulation of ACh currents for the WT. ***D*** Saz-A and ***E*** TC-2559 concentration-response curves in the absence (blue) and presence (green) of 10 μM NS9283 normalized to 1 mM ACh co-applied with 10 μM NS206 (Est $P_{o,max}$) at 3α4:2β2 receptors. Dots represent the mean ± s.e.m.

doi:10.1371/journal.pone.0161154.g003

The application of 10 μM NS206 increased the response of the receptors to either 10 μM or 1 mM ACh. When co-applied with high concentrations, NS206 typically elicited a second smaller peak when the solution was washed off (Fig 3B). This is likely due to a combination of desensitized states and the binding kinetics of both ACh and NS206. The mean peak current amplitude of ACh_{max} (1 mM) was increased by a factor of 2.0 ($n = 7$) when co-applied with NS206 for 3α4:2β2 receptor, allowing for an estimate of maximum open channel probability, while 10 μM NS206 increased the response to 10 μM ACh by nearly 4-fold (Fig 3C). Thus, the relative open probability for a saturating concentration of ACh is only half the Est. $P_{o,max}$ obtained with co-applied NS206. The *Est.* P_o response curves for the partial agonists (Saz-A and TC-2559) alone or when co-applied with NS9283 were obtained by transforming the maximum responses normalized to 1 mM ACh to an *Est.* $P_{o,max}$ (Fig 3D and 3E; Table 1). The resulting $P_{o,max}$ for Saz-A and TC-2559 that were used to calculate the values of the equilibrium constants were 9.1% and 20%, respectively, and in the presence of NS9283 these values increased to 39% and 59% ($n = 6$), respectively (Fig 3D and 3E; Table 1).

An important caveat to this approach is that the accuracy of the *Est.* P_o is dependent on response of the potentiator and ACh having an open probability of 1 [36]. Therefore, the *Est.* P_o using this method may overestimate the actual P_o of the ACh, Saz-A and TC-2559 curves if NS206 co-applied with ACh does not open all of the channels. To compensate for this possibility, we have also determined equilibrium constants where the *Est.* $P_{o,max}$ for 1 mM ACh is 0.4 and 0.45 to ensure that our conclusions are not dependent on the precise value of P_o.

Models of α4β2 Receptor Activation

To determine how binding at the α4-α4 site alters receptor transitions during activation, two simple linear kinetic models were used to describe receptor activation when two α4-β2 binding sites are occupied. For simplicity the binding of two ligands at α4-β2 sites is combined into a single step. The first model (Model 1) is a three-step model designed to determine whether the third ligand alters: (i) the binding affinity of ligands at the α4-β2 site (cooperativity); (ii) the transitions between closed and open states (gating) or (iii) the transitions between open and a distinct closed state in the presence of ligand (desensitization). The second model (Model 2) expanded the gating transitions to investigate whether a pre-activation (flipping) step is altered. We then determined which equilibrium constant was principally modulated in these models when NS9283 bound to the α4-α4 site potentiated the Saz-A or TC-2559 responses.

Model 1. The open probability for the first model is given by (Fig 4A):

$$P_o^{Ctrl} = \frac{E.[A]}{K + [A](1 + E + E.D)} \tag{1}$$

Where E is the gating constant, [A] is the agonist concentration, K is the dissociation constant of Saz-A or TC-2559, and D is the desensitizing constant. By rearranging Eq 1 to isolate [A]:

$$P_o^{Ctrl} = \frac{\frac{E}{1+E+E.D}}{1 + \frac{K}{[A].(1+E+E.D)}} \tag{2}$$

The open probability curve will fit to a Hill equation with a slope coefficient of one, since the ligand binding steps have been simplified by aggregating into a single transition. It follows from Eq 2 that the maximum open probability (when [A] approaches infinity) is given by:

$$P_{o,max}^{Ctrl} = \frac{E}{1 + E + E.D} \tag{3}$$

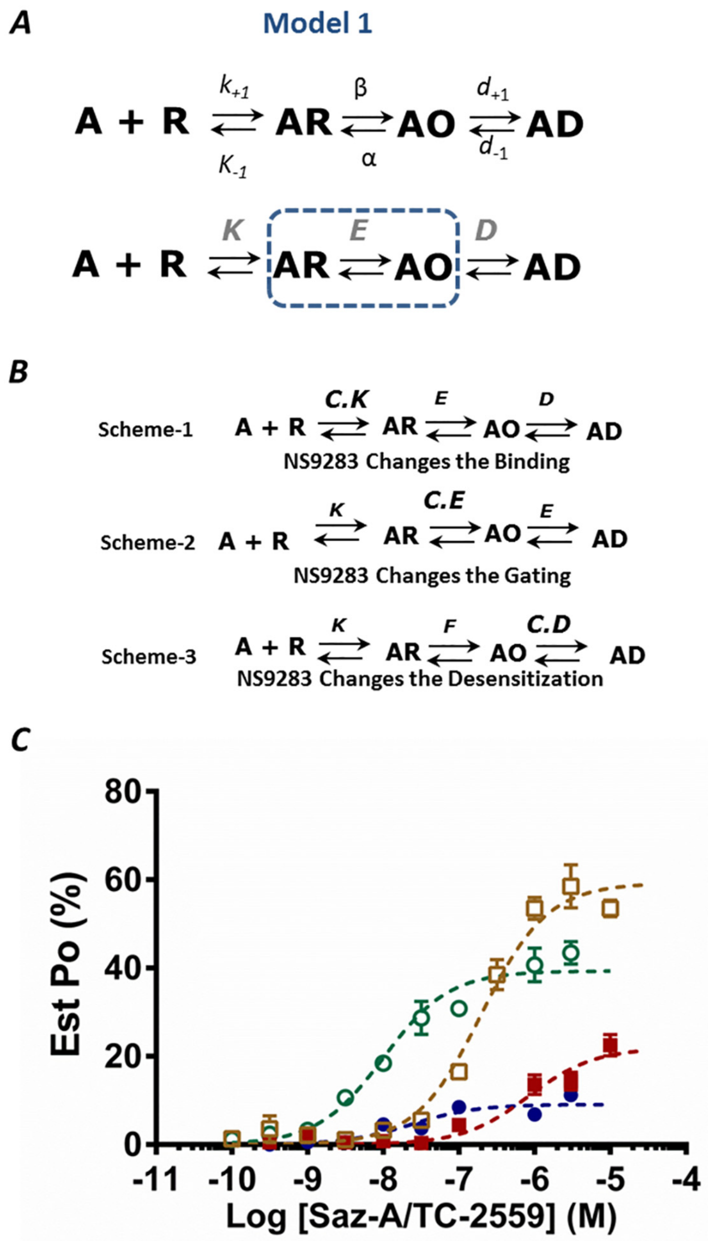

Fig 4. Model-1. A. Linear Model of receptor activation incorporating binding (AR), open (AO) and closed-desensitized (AD) states. The equilibrium constants are defined in terms of the microscopic rate constants: the ligand equilibrium dissociation constant is $K = k_{-1} / k_{+1}$; the gating equilibrium constant is $E = \beta / \alpha$; and the desensitization equilibrium constant $D = d_{+1}/d_{-1}$. **B.** Three schemes from 1–3, represent the proposed model to test whether NS9283 changes the gating (*E*), desensitization (*D*) or binding (*K*) equilibrium constants to elicit its effect. **C.** Saz-A (blue) and TC-2559 (maroon) by themselves and in the presence of NS9283 (Saz-A —Green; TC-2559—brown) were plotted (mean ± s.e.m.) (data from Fig 2E and 2F). The dotted curves represent predicted open-probability responses from the model for the control (Saz-A—Blue dotted; TC-2559 –Maroon dotted) and when gating constant (E) was multiplied by the constant C_E (Saz-A—Green dotted; TC-2559—Brown dotted).

doi:10.1371/journal.pone.0161154.g004

and the half-maximum effective concentration is:

$$EC_{50}^{Ctrl} = \frac{K}{1 + E + E.D} \tag{4}$$

Table 2. Kinetic equilibrium constants derived from three-step models proposed.

Receptor	Ligand	Model-1			Model-2			C
		K	*E*	*D*	*K*	*E*	*F*	
3α4:2β2	Saz-A	27	0.10	0.50	27	1.99	0.05	8.75
	TC-2559	833	0.27	0.28	836	3.59	0.08	9.15

K, *E*, *D* and *F* represent binding, gating, desensitized and flipping equilibrium constants respectively, as discussed in the text. Value of constant multiplier 'C' derived from the models is shown.

doi:10.1371/journal.pone.0161154.t002

It is apparent from Eq 3 that to determine the equilibrium constants for this kinetic model, the open probability (P_o) of the receptor must be estimated by normalizing to the responses to ACh and NS206, rather than normalizing to the response of a saturating concentration of ACh.

A Third Ligand Bound at the α4-α4 Site Regulates Gating Transitions in Model 1

We first hypothesized that a third ligand (NS9283) bound at the α4-α4 site, NS9283, increases the gating transitions (E) in Model 1 by a constant multiplication factor C_E (Fig 4; Scheme 1).

The open probability in the presence of NS9283 ($P^{NS}_{o.max}$; the superscript denotes when NS9283 is co-applied) and half-maximum effective concentration (EC^{NS}_{50}) can be described by substituting E with $C_E.E$ in Eqs 3 and 4 respectively. The value of C_E can then be calculated from the ratios of the EC_{50} values and the maximum open probabilities in control and when potentiated by NS9283, as follows:

$$\frac{EC^{ctrl}_{50}}{P^{ctrl}_{o,max}} = \frac{K}{E} \tag{5}$$

$$\frac{EC^{NS}_{50}}{P^{NS}_{o,max}} = \frac{K}{C_E.E} \tag{6}$$

$$C_E = \frac{P^{NS}_{o,max}}{P^{ctrl}_{o,max}} \times \frac{EC^{ctrl}_{50}}{EC^{NS}_{50}} \tag{7}$$

Using Eq 7, the values of the constant 'C_E' calculated from the change in EC_{50} and $P_{o,max}$ values when NS9283 was applied in addition to Saz-A and TC-2559 were 8.7 and 9.2 respectively (Table 2). As NS9283 binds selectively to the α4-α4 site, we assumed that it has the same multiplier effect; regardless of which partial agonist is bound at the α4-β2 site. Hence, the values of C_E for Saz-A and TC-2559 were averaged to 8.94. Equilibrium constants were then calculated using the following equations. Rearranging Eq 3:

$$\frac{1}{P^{Ctrl}_{o.max}} - 1 = \frac{1 + D.E}{E} \tag{8}$$

An equivalent expression in the presence of NS9283:

$$C_E\left(\frac{1}{P^{NS}_{o.max}} - 1\right) = \frac{1 + C_E D.E}{E} \tag{9}$$

By subtracting Eq 8 from Eq 9.

$$C_E\left(\frac{1}{P^{NS}_{o.max}}-1\right)-\left(\frac{1}{P^{Ctrl}_{o.max}}-1\right)=D.(C_E-1) \tag{10}$$

Rearranging to give D.

$$D=\frac{C_E\left(\frac{1}{P^{NS}_{o,max}}-1\right)-\left(\frac{1}{P^{ctrl}_{o,max}}-1\right)}{C_E-1} \tag{11}$$

Rearranging Eq 3 to give E.

$$E=\frac{P^{ctrl}_{o,max}}{(1-P^{ctrl}_{o,max}-D.P^{ctrl}_{o,max})} \tag{12}$$

Rearranging Eqs 5 and 6 to give K

$$K=E.\frac{EC^{ctrl}_{50}}{P^{ctrl}_{o,max}}=C_E.E.\frac{EC^{NS}_{50}}{P^{NS}_{o,max}} \tag{13}$$

The values obtained for D, E and K (Table 2) were substituted into Eq 1 to predict the P_o at a given concentration of Saz-A or TC-2559. The predicted P_o response curve for both Saz-A and TC-2559 co-applied with NS9283 was derived by substituting the same values into Eq 1, with the exception that E was substituted with $C_E.E$. The predicted P_o response curves describe the experimentally derived data points well (Fig 4C). Further, when the values for D, E and K were determined using an *Est.* P_o for 1 mM ACh of 0.4 or 0.45 and substituted into Eq 1, the predicted P_o response curves also describe the experimentally derived data points well, demonstrating that the exact value for P_o is not necessary (S1 Fig).

We next explored the hypothesis that NS9283 decreases the desensitization constant (D) by a constant factor C_D (Fig 4B; Scheme 2). The maximum open probability and the EC_{50} for this scheme are:

$$P^{NS}_{o,max}=\frac{E}{1+E+E.C_D.D} \tag{14}$$

$$EC^{NS}_{50}=\frac{K}{1+E+E.C_D.D} \tag{15}$$

The ratio of EC_{50} and $P_{o,max}$ values gives:

$$\frac{EC^{ctrl}_{50}}{P^{ctrl}_{o,max}}=\frac{EC^{NS}_{50}}{P^{NS}_{o,max}}=\frac{K}{E} \tag{16}$$

This relationship is not supported by the experimental data, where NS9283 causes a decrease in the EC_{50} value and an increase in the $P_{o,max}$. Thus, the ratio of EC^{NS}_{50} and $P^{NS}_{o,max}$ values will be smaller than in control. With Saz-A the ratio in control is 253 nM, but only 29 nM when potentiated by NS9283, while with TC-2559 the ratio is 3.1 μM, but 336 nM when potentiated by NS9283. Hence, it is unlikely that NS9283 elicits its action by a decrease in desensitization. Finally, we investigated whether NS9283 binding to the α4-α4 site alters the binding equilibrium (K) of Saz-A or TC-2559 by a constant factor C_K (Fig 4B; Scheme 3). However, the $P_{o,max}$ does not depend on the value of K (Eq 3) and is predicted to remain unchanged when K is modulated. This is inconsistent with the experimental data and was rejected as a possibility.

Taken together, this model predicts that a third ligand increases gating transitions from the closed to the open state to potentiate receptor function, primarily increasing the efficacy of the response. However, the gating step in this model is likely to be an oversimplification of the actual gating transitions. Transitions into a pre-activated intermediate state has been essential to explain the efficacy of partial agonists and the actions of modulators at other LGICs [27,28,30]; Hence we chose to test whether transitions into a pre-activated state were altered when NS9283 was bound at the α-α interface.

Model 2. This model expanded the gating step from Model 1 to include a pre-activated closed state between the ligand-bound and open states and hence serves as an extension of Model 1 (Fig 5).

Since desensitization was found to be unnecessary to describe the action of NS9283 in Model 1, it was not included in this model for simplicity. Therefore, Model 2 is a three-step model with a binding, pre-activated and gating step equivalent to that used by Gielen *et al* (2012) to describe the potentiation of $GABA_A$Rs by diazepam. The open probability for this model is given by the following:

$$P_o = \frac{E.F.[A]}{K + [A](1 + F + E.F)} \tag{17}$$

Where K is the dissociation constant, F is the pre-activating equilibrium constant and E is the gating constant. It also follows that the maximum open probability is given by:

$$P_{o,max} = \frac{E.F}{1 + F + E.F} \tag{18}$$

Thus, both F and E will have an influence on the observed macroscopic efficacy. The half-maximum effective concentration is:

$$EC_{50} = \frac{K}{1 + F + E.F} \tag{19}$$

A Third Ligand Bound at the α4-α4 site Regulates Pre-Activating Transitions in Model 2

We investigated the possibility that NS9283 increases the pre-activation constant, F, by a factor, C_F, in Model 2 (Fig 5B; Scheme 2). By substituting F with $C_F.F$ in Eqs 18 and 19, the value of C_F can be determined numerically from the equation:

$$C_F = \frac{EC_{50}^{ctrl}}{EC_{50}^{NS}} \times \frac{P_{o,max}^{NS}}{P_{o,max}^{ctrl}} \tag{20}$$

The equilibrium constants K, F and E can then be calculated using the following equations (Gielen et al., 2012). Rearranging Eq 18:

$$\frac{1}{P_{o.max}^{Ctrl}} - 1 = \frac{1 + F}{E.F} \tag{21}$$

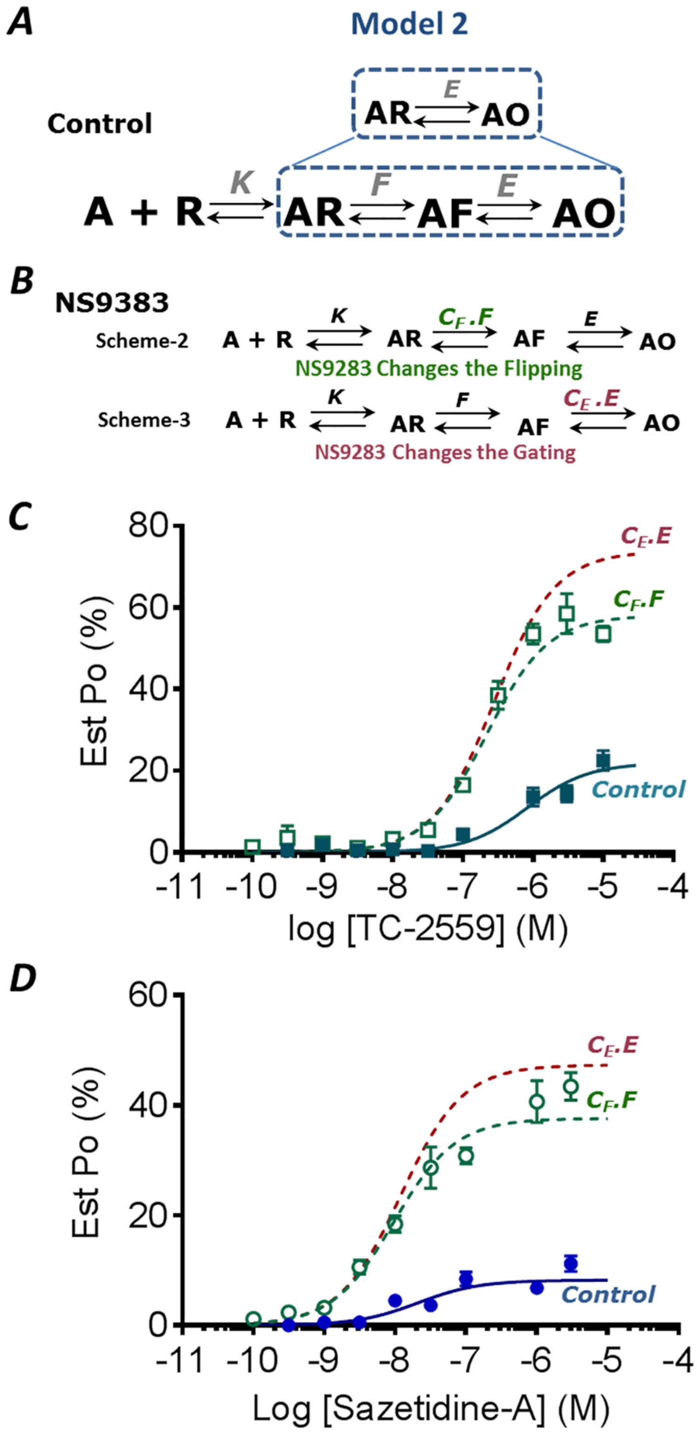

Fig 5. Model-2. A. Linear Model of receptor activation incorporating an intermediate flip state (AF) between ligand bound (AR) and open (AO) states. Dotted box suggests that the gating step from Fig 4 is extended to include flipping step in this model. **B.** Proposed schemes represent when NS9283 changes the pre-activation (*F*) or gating (*E*) constants. The equilibrium constants were estimated using Scheme 2. All equilibrium constants are defined as previously, and F is equal to the forward rate/reverse rate. **C** TC-2559 and **D** Saz-A data plotted (mean ± s.e.m.) is same as Fig 4, while the dotted curves represent predicted open-probability curves from Models 2 where either the pre-activation constant (*F* green) or the gating constant (*E* red) were multiplied by the constant C_F or C_E respectively.

doi:10.1371/journal.pone.0161154.g005

An equivalent expression in the presence of NS9283:

$$C_F\left(\frac{1}{\mathrm{P}^{NS}_{o.max}}-1\right)=\frac{1+C_F F}{E.F} \tag{22}$$

By subtracting Eq 21 from Eq 22 and re-arranging in terms of E:

$$E=\frac{C_F-1}{C_F\left(\frac{1}{P^{\mathrm{NS}}_{\mathrm{o,max}}}-1\right)-\left(\frac{1}{P^{\mathrm{ctrl}}_{\mathrm{o,max}}}-1\right)} \tag{23}$$

The value of E can then be calculated using Eq 23. F was calculated by rearranging Eq 18, similar to Eq 12 above, and K can then be calculated by substituting C_F into Eq 13 and multiplying the right-hand side by F. From Eq 20, the value of C_F was calculated for NS9283 potentiation of Saz-A and TC-2559 activation was calculated, averaged to 8.94 (as discussed in Model 1) and used to determine the values for E, F and K (Table 2). These values were then used to predict the P_o from Eq 17 for a given concentration of agonist for Saz-A and TC-2559 with and without NS9283. The predicted P_o response curves correspond well with the experimental data (Fig 5C and 5D). Thus, the mechanism of NS9283 potentiation of Saz-A and TC-2559 could be refined by an increase in the equilibrium of the pre-activating step (Fig 4C and 4D).

The final hypothesis to be tested was that NS9283 increases the gating constant E of Model 2 by the constant C_E (Fig 5B; Scheme 3). Modulation of E is predicted to be affect both the maximum response, $P^{\mathrm{NS}}_{\mathrm{o,max}}$, and the half-maximum concentration, $\mathrm{EC}^{\mathrm{NS}}_{50}$. The value of C_E can be calculated as before from Eq 17, and the average value of 8.94 was used. The potentiation of the EC_{50} and $P_{\mathrm{o,max}}$ are described by:

$$\mathrm{EC}^{\mathrm{NS}}_{50}=\frac{\mathrm{EC}^{\mathrm{ctrl}}_{50}}{1+(C_\mathrm{E}-1).P^{\mathrm{ctrl}}_{\mathrm{o,max}}} \tag{24}$$

$$P^{\mathrm{NS}}_{\mathrm{o,max}}=\frac{C_\mathrm{E}.P^{\mathrm{ctrl}}_{\mathrm{o,max}}}{1+(C_\mathrm{E}-1).P^{\mathrm{ctrl}}_{\mathrm{o,max}}} \tag{25}$$

which shows that the EC_{50} and $P_{\mathrm{o,max}}$ of the NS9283 curves are dependent only on the values of C_E, the agonist potency (EC_{50}) and the $P_{\mathrm{o,max}}$ and not on the specific values of E, F and K (Table 2). Notably, the same equations would be derived for a simple Castillo-Katz mechanism with a single binding step and gating transition. The predicted relative response curves describe a shift in the EC_{50} and $P_{\mathrm{o,max}}$ with NS9283 potentiation, with predicted EC_{50} values of 13 nM and 140 nM, similar to the measured values of 11 nM and 195 nM for Saz-A and TC-2559, respectively. However, the maximum response overshoots the experimental data, predicting a $P_{\mathrm{o,max}}$ of 47% and 69% in comparison to the measured values of 39% and 59% for Saz-A and TC-2559, respectively (Fig 4C and 4D). Further, when the values for K, F and E were determined using an *Est.* P_o for 1 mM ACh of 0.4 or 0.45 and substituted into Eq 17, the predicted P_o response curves also describe the experimentally derived data points well when the pre-activated constant F, rather than the gating constant E, was altered (S1 Fig).

Taken together, this suggests that the increase in gating transitions observed in Model 1 is primarily due to an increase in transitions into the pre-activated state, with the gating transitions unchanged, when the third α4-α4 site is occupied to primarily increase the efficacy of the response.

Discussion

Here, we demonstrate that binding of an agonist to the α4-α4 binding site of the 3α4:2β2 receptor increases the equilibrium constant favouring transitions to the pre-activated, 'flip' state. We used site-selective ligands coupled with two distinct linear kinetic models to interrogate how the activation mechanism is altered when the third α4-α4 agonist-binding site is occupied in addition to the two α4-β2 sites.

When NS9283 bound to the α4-α4 site, it enhanced both the potency and efficacy of Saz-A or TC-2559 activation via the α4-β2 sites, and the concentration-response curves could be described by the alteration of just one transition in our kinetic models. Both these models indicate that when the third α4-α4 site is occupied by NS9283, the equilibrium constants defining the transitions from the closed to the open states are increased. Further, the second model incorporating a pre-activated step indicates more specifically that the transitions to the pre-activated state are increased. Although it would be ideal to obtain $P_{o,max}$ from single channel recordings, the changes in the *Est.* $P_{o,max}$ did not make any differences to the conclusions that were reached and a similar method to estimate open probability with an allosteric modulator, has been used for other cys-loop receptors to compare the magnitude of an allosteric shift between different receptor subtypes [35,36].

The α4β2 nAChR is considered to be an important target for treating schizophrenia, attention deficit hyperactivity disorder (ADHD) and pain; and several drugs have been shown to increase pro-cognitive and analgesic effects in pre-clinical as well as clinical trials [3–6]. However, only one drug, varenicline, has come to the market in recent years that is used for smoking cessation [37]. A key aspect of drug development is the selectivity for specific receptor subtypes that is complicated by the fact that α4β2 exists in two different stoichiometries, the 2α4:3β2 and 3α4:2β2. We and others have shown that the presence of an α4-α4 agonist interface in the 3α4:2β2, in addition to the α4-β2 interface present in both stoichiometries, greatly change the pharmacological properties of the receptor [11,17,18]. Because of this additional agonist binding site, several agonists (e.g. ACh, epibatidine), partial-agonists (e.g. NS3573), antagonists (e.g. methyllycaconitine) and modulators (e.g. Zn^{2+}) act differently on the two stoichiometries [15,18,19,38]. Further, ligands that selectively act on the α4-β2 and the α4-α4 agonist binding sites like Saz-A [8] and NS9283 [19,34] respectively, provide us with a great tool to tease out signal transduction initiated from specific agonist binding sites, along with serving as lead molecules in structure activity relationship (SAR) studies that enable us to target the two stoichiometries more specifically.

While traditionally positive allosteric modulators have been defined as ligands that increase the response of the native agonist without activating the receptor themselves, NS9283 binds to the same site as the native agonist ACh. This has recently led NS9283 to be referred to as either an agonist [39], or a site-selective agonist [20] rather than as a positive allosteric modulator. Mechanistically NS9283 is behaving like an agonist, but cannot activate receptor when only one binding site is occupied. Targeting such a site can present advantages for therapeutics, as compounds that bind at these sites only increase the response to the native ligand, rather than activate all available receptors, potentially reducing the side-effects of the drug [40].

Rather than being a property of the closed to open transition as previously supposed, partial agonism in the Cys-loop receptor family can be ascribed to intermediate preactivation step that has been termed a 'flip' or a 'prime' state [27,28,41,42]. Single-channel recordings fitted with mechanisms that include a pre-activated state have demonstrated that the low efficacy activation of Cys-Loop receptors such as GlyRs by taurine, the muscle nAChR by tetramethylammonium [27] and 5-HT_{3A}Rs by tryptamine [42] is determined by the affinity of the ligand to the pre-activated states. These discoveries that the efficacy of an agonist is in large part determined

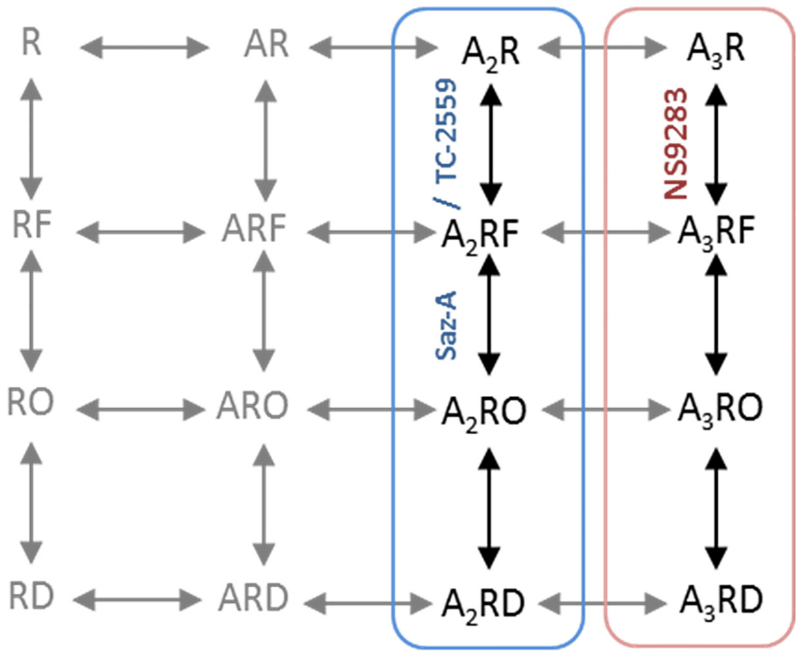

Fig 6. Proposed model. A schematic of proposed receptor model for the activation of WT 3α4:2β2 nAChR. The model includes unbound (R), one agonist bound (AR), two agonist bound (A_2R) and three agonist bound (A_3R) receptor states. Intermediate 'flip' state (RF), Open state (RO) and desensitized state (RD) are shown. Blue frame includes receptor activation model for agonists (eg. Saz-A and TC-2559) that selective bind at the α4-β2 interface and red frame shows receptor activation for ligands selective for α4-α4 interface (NS9283). Dashed lines and grey states indicate transitions and states not discretely tested in our experiments but are considered likely to exist.

doi:10.1371/journal.pone.0161154.g006

by perturbations of a pre-activated state thus have implications for drug development. Ligands that are targeted to the interface of extracellular domains, such as diazepam and NS9283, are likely to potentiate the response of the native agonist through favouring transitions into the pre-activated state. This will manifest itself primarily through an increase in both potency and efficacy of the native agonist, but the magnitude of these increases will be dependent on the intrinsic efficacy of the agonist. The combined knowledge of the intrinsic efficacy of the native ligand, the binding sites that distinct stoichiometries make available and the likely mechanism of action of a ligand binding at putative binding site can then be used in the early stages of drug development to predict the pharmacological effects of a drug at the receptors of interest.

With this understanding of how the binding of a third agonist at the α4-α4 site, modulates receptor activity, we can also propose a plausible kinetic model of ACh activation for the 3α4:2β2 receptor (Fig 6). In this scheme, the binding of two molecules of ACh at the α4-β2 site, similar to Saz-A or TC-2559, would elicit a conformational change of the receptor into a pre-activated state, whereby the receptor conformation can then transition into the open state. When the third molecule of ACh binds to the α4-α4 site, similar to NS9283, the conformational change of the receptor into the pre-activated state becomes much more likely, driving an increased probability of the receptor being in the open state. When measuring macroscopic responses, the combination of the higher binding affinity of ACh at the α4-β2 binding sites, and the increased efficacy when three binding sites are occupied, leads to biphasic concentration-response curve where ACh is essentially a partial agonist at low concentrations. After prolonged periods in the open state, the receptor transitions to a desensitized state that is resistant to activation by agonist binding. This pre-activated state described in the current study can

well be a representation of multiple intermediate states that make the 'conformational wave' that is represented in a single state.

This model is supported by activation mechanisms of other LGICs, including the glycine receptor (GlyR), where the pre-activated state has been detected in single channel recordings [27]. In these models, both the nature of the agonist and the number of agonist molecules bound alter transitions into the pre-activated states, while the transition into the final gating step is a fast, stereotyped reaction that has little dependence on the nature of the agonist [27,41]. We did not consider channel openings from un-liganded and mono-liganded receptors in our linear kinetic model, and without measurements from single channel recordings we can only speculate on their importance. Undoubtedly these states exist, albeit extremely rarely, and we cannot make any conclusions on their relevance from macroscopic recordings [43,44].

Ideally, we would be able to directly test this model of ACh-activation by fitting data generated from single-channel recordings to our scheme and estimating the equilibrium rate constants for kinetic schemes. However, measuring single-channel recordings from α4β2 nAChRs is problematic due to channel rundown and desensitization, which prevents reliable estimates of the open probability (P_o), and the values of equilibrium rate constants from being derived [45]. Despite this, simulations using macroscopic data combined with simple kinetic models can be a useful tool to understand allosteric receptor activation, even considering for the limitations of modelling macroscopic data where receptors are in different conformational states at any given time during agonist application [25,46]. This is best exemplified by the discovery that benzodiazepines modulate transitions into the pre-activated state at γ-aminobutyric acid ($GABA_A$Rs) using macroscopic data from *Xenopus oocyte* electrophysiology [30].

In summary, we have proposed and tested plausible activation models for the 3α4:2β2 nAChR isoform. By systematically analysing macroscopic data with two models, we demonstrate that when a third agonist molecule is bound at the α-α interface, transitions into the pre-activated state are favoured to increase the activation levels, and ultimately the efficacy of the response. Considering the widespread clinical use of benzodiazepines, this discovery of a conserved mechanism that benzodiazepines and ACh potentiate receptor activation *via* a third binding site can be exploited to develop therapeutics with similar properties at other cys-loop receptors.

Supporting Information

S1 Fig. Effect of Changes in *Est* P_{omax} on concentration-response relationships derived from both **A** the desensitizing (Model 1) and **B** the pre-activated model (Model 2). Peak currents to Saz-A (●) and without (○) 10 μM NS-9283, and to TC-2559 with (■) and without (□) 10 μM NS9283 were normalized to an *Est* P_{omax} for 1 mM ACh of 0.4 (left) and 0.45 (right). Dashed lines represent the simulated curves from A Model 1 where NS9283 alters the gating constant E, and **B** Model 2 where either the pre-activated constant F is altered ($C_F.F$) or the gating constant ($C_E.E$) is altered by NS9283.
(PDF)

Acknowledgments

This work was funded by the National Health and Medical Research Council of Australia (NHMRC) Project Grant 1069417. DCI is funded by an International Postgraduate Research Scholarship by the University of Sydney and received support of a John Lamberton Scholarship. PKA was co-funded by Imk Almene Fond, Denmark.

Author Contributions

Conceived and designed the experiments: DI TML PKA TB MC NA.

Performed the experiments: DI.

Analyzed the data: DI TML NA.

Contributed reagents/materials/analysis tools: DI TML PKA TB NA.

Wrote the paper: DI TML PKA TB MC NA.

References

1. Engelman HS, MacDermott AB (2004) Presynaptic ionotropic receptors and control of transmitter release. Nature Reviews Neuroscience 5: 135–145. PMID: 14735116
2. Paterson D, Nordberg A (2000) Neuronal nicotinic receptors in the human brain. Progress in Neurobiology 61: 75–111. PMID: 10759066
3. Rezvani AH, Levin ED (2001) Cognitive effects of nicotine. Biological Psychiatry 49: 258–267. PMID: 11230877
4. Leonard S, Mexal S, Berger R, Olincy A, Freedman R (2007) Smoking and schizophrenia: Evidence for self medication. Schizophrenia Bulletin 33: 262–263.
5. Bain EE, Robieson W, Pritchett Y, Garimella T, Abi-Saab W, et al. (2013) A Randomized, Double-Blind, Placebo-Controlled Phase 2 Study of alpha 4 beta 2 Agonist ABT-894 in Adults with ADHD. Neuropsychopharmacology 38: 405–413. doi: 10.1038/npp.2012.194 PMID: 23032073
6. Timmermann DB, Sandager-Nielsen K, Dyhring T, Smith M, Jacobsen AM, et al. (2012) Augmentation of cognitive function by NS9283, a stoichiometry-dependent positive allosteric modulator of alpha 2- and alpha 4-containing nicotinic acetylcholine receptors. British Journal of Pharmacology 167: 164–182. doi: 10.1111/j.1476-5381.2012.01989.x PMID: 22506660
7. Nelson ME, Kuryatov A, Choi CH, Zhou Y, Lindstrom J (2003) Alternate stoichiometries of alpha4beta2 nicotinic acetylcholine receptors. Molecular Pharmacology 63: 332–341. PMID: 12527804
8. Zwart R, Carbone AL, Moroni M, Bermudez I, Mogg AJ, et al. (2008) Sazetidine-A is a potent and selective agonist at native and recombinant alpha 4 beta 2 nicotinic acetylcholine receptors. Molecular Pharmacology 73: 1838–1843. doi: 10.1124/mol.108.045104 PMID: 18367540
9. Carbone AL, Moroni M, Groot-Kormelink PJ, Bermudez I (2009) Pentameric concatenated (alpha 4)(2)(beta 2)(3) and (alpha 4)(3)(beta 2)(2) nicotinic acetylcholine receptors: subunit arrangement determines functional expression. British Journal of Pharmacology 156: 970–981. doi: 10.1111/j.1476-5381.2008.00104.x PMID: 19366353
10. Benallegue N, Mazzaferro S, Alcaino C, Bermudez I (2013) The additional ACh binding site at the alpha 4(+)/alpha 4(-) interface of the (alpha 4 beta 2)(2)alpha 4 nicotinic ACh receptor contributes to desensitization. British Journal of Pharmacology 170: 304–316. doi: 10.1111/bph.12268 PMID: 23742319
11. Ahring PK, Olsen JA, Nielsen EO, Peters D, Pedersen MHF, et al. (2015) Engineered alpha 4 beta 2 nicotinic acetylcholine receptors as models for measuring agonist binding and effect at the orthosteric low-affinity alpha 4-alpha 4 interface. Neuropharmacology 92: 135–145. doi: 10.1016/j.neuropharm.2014.12.035 PMID: 25595102
12. Tapia L, Kuryatov A, Lindstrom J (2007) Ca2+ permeability of the (alpha 4)(3)(beta 2)(2) stoichiometry greatly exceeds that of (alpha 4)(2)(beta 2)(3) human acetylcholine receptors. Molecular Pharmacology 71: 769–776. PMID: 17132685
13. Kuryatov A, Luo J, Cooper J, Lindstrom J (2005) Nicotine acts as a pharmacological chaperone to up-regulate human alpha 4 beta 2 acetylcholine receptors. Molecular Pharmacology 68: 1839–1851. PMID: 16183856
14. Mazzaferro S, Gasparri F, New K, Alcaino C, Faundez M, et al. (2014) Non-equivalent ligand selectivity of agonist sites in (alpha4beta2)2alpha4 nicotinic acetylcholine receptors: a key determinant of agonist efficacy. J Biol Chem 289: 21795–21806. doi: 10.1074/jbc.M114.555136 PMID: 24936069
15. Absalom NL, Quek G, Lewis TM, Qudah T, von Arenstorff I, et al. (2013) Covalent Trapping of Methyllycaconitine at the alpha 4-alpha 4 Interface of the alpha 4 beta 2 Nicotinic Acetylcholine Receptor ANTAGONIST BINDING SITE AND MODE OF RECEPTOR INHIBITION REVEALED. Journal of Biological Chemistry 288: 26521–26532. doi: 10.1074/jbc.M113.475053 PMID: 23893416

16. Moroni M, Zwart R, Sher E, Cassels BK, Bermudez I (2006) alpha4beta2 nicotinic receptors with high and low acetylcholine sensitivity: pharmacology, stoichiometry, and sensitivity to long-term exposure to nicotine. Molecular Pharmacology 70: 755–768. PMID: 16720757

17. Harpsoe K, Ahring PK, Christensen JK, Jensen ML, Peters D, et al. (2011) Unraveling the high- and low-sensitivity agonist responses of nicotinic acetylcholine receptors. J Neurosci 31: 10759–10766. doi: 10.1523/JNEUROSCI.1509-11.2011 PMID: 21795528

18. Mazzaferro S, Benallegue N, Carbone A, Gasparri F, Vijayan R, et al. (2011) Additional acetylcholine (ACh) binding site at alpha4/alpha4 interface of (alpha4beta2)2alpha4 nicotinic receptor influences agonist sensitivity. J Biol Chem 286: 31043–31054. doi: 10.1074/jbc.M111.262014 PMID: 21757735

19. Grupe M, Jensen AA, Ahring PK, Christensen JK, Grunnet M (2013) Unravelling the mechanism of action of NS9283, a positive allosteric modulator of (alpha 4)(3)(beta 2)(2) nicotinic ACh receptors. British Journal of Pharmacology 168: 2000–2010. doi: 10.1111/bph.12095 PMID: 23278456

20. Olsen JA, Ahring PK, Kastrup JS, Gajhede M, Balle T (2014) Structural and functional studies of the modulator NS9283 reveal agonist-like mechanism of action at alpha4beta2 nicotinic acetylcholine receptors. J Biol Chem 289: 24911–24921. doi: 10.1074/jbc.M114.568097 PMID: 24982426

21. Shahsavar A, Ahring PK, Olsen JA, Krintel C, Kastrup JS, et al. (2015) Acetylcholine-Binding Protein Engineered to Mimic the alpha4-alpha4 Binding Pocket in alpha4beta2 Nicotinic Acetylcholine Receptors Reveals Interface Specific Interactions Important for Binding and Activity. Molecular Pharmacology 88: 697–707. doi: 10.1124/mol.115.098061 PMID: 26180047

22. Colquhoun D (1998) Binding, gating, affinity and efficacy: The interpretation of structure-activity relationships for agonists and of the effects of mutating receptors. British Journal of Pharmacology 125: 924–947. PMID: 9846630

23. Grosman C, Zhou M, Auerbach A (2000) Mapping the conformational wave of acetylcholine receptor channel gating. Nature 403: 773–776. PMID: 10693806

24. Delcastillo J, Katz B (1957) Interaction at End-Plate Receptors between Different Choline Derivatives. Proceedings of the Royal Society Series B-Biological Sciences 146: 369–380.

25. Jones MV, Westbrook GL (1996) The impact of receptor desensitization on fast synaptic transmission. Trends in Neurosciences 19: 96–101. PMID: 9054063

26. Akasu T, Ohta Y, Koketsu K (1984) Neuropeptides Facilitate the Desensitization of Nicotinic Acetylcholine-Receptor in Frog Skeletal-Muscle Endplate. Brain Research 290: 342–347. PMID: 6198044

27. Lape R, Colquhoun D, Sivilotti LG (2008) On the nature of partial agonism in the nicotinic receptor superfamily. Nature 454: 722–727. doi: 10.1038/nature07139 PMID: 18633353

28. Mukhtasimova N, Lee WY, Wang HL, Sine SM (2009) Detection and trapping of intermediate states priming nicotinic receptor channel opening. Nature 459: 451–U172. doi: 10.1038/nature07923 PMID: 19339970

29. Williams DK, Wang J, Papke RL (2011) Investigation of the molecular mechanism of the alpha7 nicotinic acetylcholine receptor positive allosteric modulator PNU-120596 provides evidence for two distinct desensitized states. Mol Pharmacol 80: 1013–1032. doi: 10.1124/mol.111.074302 PMID: 21885620

30. Gielen MC, Lumb MJ, Smart TG (2012) Benzodiazepines Modulate GABA(A) Receptors by Regulating the Preactivation Step after GABA Binding. Journal of Neuroscience 32: 5707–5715. doi: 10.1523/JNEUROSCI.5663-11.2012 PMID: 22539833

31. Timmermann DB, Gronlien JH, Kohlhaas KL, Nielsen EO, Dam E, et al. (2007) An allosteric modulator of the alpha7 nicotinic acetylcholine receptor possessing cognition-enhancing properties in vivo. J Pharmacol Exp Ther 323: 294–307. PMID: 17625074

32. Wang JY, Kuryatov A, Jin Z, Norleans J, Kamenecka TM, et al. (2015) A Novel alpha 2/alpha 4 Subtype-selective Positive Allosteric Modulator of Nicotinic Acetylcholine Receptors Acting from the C-tail of an alpha Subunit. Journal of Biological Chemistry 290: 28834–28846. doi: 10.1074/jbc.M115.676551 PMID: 26432642

33. Li P, Steinbach JH (2010) The neuronal nicotinic alpha 4 beta 2 receptor has a high maximal probability of being open. British Journal of Pharmacology 160: 1906–1915. doi: 10.1111/j.1476-5381.2010.00761.x PMID: 20649589

34. Olsen JA, Kastrup JS, Peters D, Gajhede M, Balle T, et al. (2013) Two Distinct Allosteric Binding Sites at alpha 4 beta 2 Nicotinic Acetylcholine Receptors Revealed by NS206 and NS9283 Give Unique Insights to Binding Activity-associated Linkage at Cys-loop Receptors. Journal of Biological Chemistry 288: 35997–36006. doi: 10.1074/jbc.M113.498618 PMID: 24169695

35. Rusch D, Zhong HJ, Forman SA (2004) Gating allosterism at a single class of etomidate sites on alpha (1)beta(2)gamma(2L) GABA(A) receptors accounts for both direct activation and agonist modulation. Journal of Biological Chemistry 279: 20982–20992. PMID: 15016806

36. Eaton MM, Bracamontes J, Shu HJ, Li P, Mennerick S, et al. (2014) gamma-aminobutyric acid type A alpha4, beta2, and delta subunits assemble to produce more than one functionally distinct receptor type. Mol Pharmacol 86: 647–656. doi: 10.1124/mol.114.094813 PMID: 25238745

37. Jorenby DE (2006) Efficacy of varenicline, an alpha 4 beta 2 nicotinic acetylcholine receptor partial agonist, vs placebo or sustained-release bupropion for smoking cessation: A randomized controlled trial (vol 296, pg 56, 2006). Jama-Journal of the American Medical Association 296: 1355–1355.

38. Moroni M, Vijayan R, Carbone A, Zwart R, Biggin PC, et al. (2008) Non-agonist-binding subunit interfaces confer distinct functional signatures to the alternate stoichiometries of the alpha 4 beta 2 nicotinic receptor: An alpha 4-alpha 4 interface is required for Zn2+ potentiation. Journal of Neuroscience 28: 6884–6894. doi: 10.1523/JNEUROSCI.1228-08.2008 PMID: 18596163

39. Wang J, Kuryatov A, Sriram A, Jin Z, Kamenecka TM, et al. (2015) An Accessory Agonist Binding Site Promotes Activation of alpha4beta2* Nicotinic Acetylcholine Receptors. J Biol Chem 290: 13907–13918. doi: 10.1074/jbc.M115.646786 PMID: 25869137

40. Williams DK, Wang J, Papke RL (2011) Positive allosteric modulators as an approach to nicotinic acetylcholine receptor-targeted therapeutics: advantages and limitations. Biochem Pharmacol 82: 915–930. doi: 10.1016/j.bcp.2011.05.001 PMID: 21575610

41. Colquhoun D, Lape R (2012) Perspectives on: conformational coupling in ion channels: allosteric coupling in ligand-gated ion channels. J Gen Physiol 140: 599–612. doi: 10.1085/jgp.201210844 PMID: 23183696

42. Corradi J, Bouzat C (2014) Unraveling mechanisms underlying partial agonism in 5-HT3A receptors. J Neurosci 34: 16865–16876. doi: 10.1523/JNEUROSCI.1970-14.2014 PMID: 25505338

43. Purohit P, Auerbach A (2009) Unliganded gating of acetylcholine receptor channels. Proc Natl Acad Sci U S A 106: 115–120. doi: 10.1073/pnas.0809272106 PMID: 19114650

44. Andersen N, Corradi J, Bartos M, Sine SM, Bouzat C (2011) Functional Relationships between Agonist Binding Sites and Coupling Regions of Homomeric Cys-Loop Receptors. Journal of Neuroscience 31: 3662–3669. doi: 10.1523/JNEUROSCI.5940-10.2011 PMID: 21389221

45. Mike A, Castro NG, Albuquerque EX (2000) Choline and acetylcholine have similar kinetic properties of activation and desensitization on the alpha 7 nicotinic receptors in rat hippocampal neurons. Brain Research 882: 155–168. PMID: 11056195

46. Papke RL (2010) Tricks of Perspective: Insights and Limitations to the Study of Macroscopic Currents for the Analysis of nAChR Activation and Desensitization. Journal of Molecular Neuroscience 40: 77–86. doi: 10.1007/s12031-009-9261-0 PMID: 19672724
